# Appraisal of a *Leishmania major* Strain Stably Expressing mCherry Fluorescent Protein for Both *In Vitro* and *In Vivo* Studies of Potential Drugs and Vaccine against Cutaneous Leishmaniasis

**DOI:** 10.1371/journal.pntd.0001927

**Published:** 2012-11-29

**Authors:** Estefania Calvo-Álvarez, Nestor Adrian Guerrero, Raquel Álvarez-Velilla, Christopher Fernández Prada, Jose María Requena, Carmen Punzón, Miguel Ángel Llamas, Francisco J. Arévalo, Luis Rivas, Manuel Fresno, Yolanda Pérez-Pertejo, Rafael Balaña-Fouce, Rosa M. Reguera

**Affiliations:** 1 Departamento de Ciencias Biomédicas, Universidad de León, León, Spain; 2 Centro de Biología Molecular “Severo Ochoa”, Universidad Autónoma de Madrid, Madrid, Spain; 3 Diomune, Parque Cientifico de Madrid, Madrid, Spain; 4 Centro de Investigaciones Biológicas, Madrid, Spain; Institut Pasteur, France

## Abstract

**Background:**

*Leishmania major* cutaneous leishmaniasis is an infectious zoonotic disease. It is produced by a digenetic parasite, which resides in the phagolysosomal compartment of different mammalian macrophage populations. There is an urgent need to develop new therapies (drugs) against this neglected disease that hits developing countries. The main goal of this work is to establish an easier and cheaper tool of choice for real-time monitoring of the establishment and progression of this pathology either in BALB/c mice or *in vitro* assays. To validate this new technique we vaccinated mice with an attenuated Δhsp70-II strain of *Leishmania* to assess protection against this disease.

**Methodology:**

We engineered a transgenic *L. major* strain expressing the mCherry red-fluorescent protein for real-time monitoring of the parasitic load. This is achieved via measurement of fluorescence emission, allowing a weekly record of the footpads over eight weeks after the inoculation of BALB/c mice.

**Results:**

*In vitro* results show a linear correlation between the number of parasites and fluorescence emission over a range of four logs. The minimum number of parasites (amastigote isolated from lesion) detected by their fluorescent phenotype was 10,000. The effect of antileishmanial drugs against mCherry+*L. major* infecting peritoneal macrophages were evaluated by direct assay of fluorescence emission, with IC_50_ values of 0.12, 0.56 and 9.20 µM for amphotericin B, miltefosine and paromomycin, respectively. An experimental vaccination trial based on the protection conferred by an attenuated Δhsp70-II mutant of *Leishmania* was used to validate the suitability of this technique *in vivo*.

**Conclusions:**

A *Leishmania major* strain expressing mCherry red-fluorescent protein enables the monitoring of parasitic load via measurement of fluorescence emission. This approach allows a simpler, faster, non-invasive and cost-effective technique to assess the clinical progression of the infection after drug or vaccine therapy.

## Author Summary

Leishmaniasis is a parasitic disease that is far from eradication. The lack of an efficacious vaccine and treatment failures are major factors in its intractable worldwide prevalence. A non-invasive imaging technique using genetically engineered parasites that expressed fluorescent proteins could give to researchers a quantitative and visual tool to characterize the parasite burden in experimental infections. In addition, it can be useful for determining the efficacy of candidate vaccines or drugs using High Throughput Screening methods that allow the testing of libraries of compounds in an automated 96-well plate format. Herein, we demonstrate that there is a good correlation between fluorescence emission and the parasite load, thus permitting the use of this output to monitor the progression of the disease. In order to validate this tool we have immunized mice prior the parasite challenge with a red-emitting parasite strain, confirming the scientific suitability of this approach as a valuable alternative model.

## Introduction

*Leishmania major* is the main cause of cutaneous leishmaniasis (CL) in the Old World. Parasites are transmitted by *Phlebotominae* sandflies whilst blood feeding on infected mammalian hosts. CL is widely spread in the developing world, affecting people in 88 countries with 1.5 million new cases reported each year. CL usually produces ulcers on the exposed parts of the body that often leave disfiguring scars, which in turn, can cause serious social prejudice [1].

Conventional *in vivo* animal models for the study of parasite-host relationships involve large number of animals. These animals are required to be slaughtered at different time points in order to identify both anatomical distribution and parasite numbers in organs and tissues. Furthermore, this approach has some important limitations that must be overcome: i) post-mortem analysis of animals makes it impossible to track the space/time progression of the pathogen within the hosts; ii) spread of the pathogen to unexpected anatomic sites can remain undetected; iii) in order to achieve precise and relevant data, it is necessary to kill large numbers of animals. Recent real-time *in-vivo* imaging techniques with genetically modified pathogens represent a valuable complementary tool. They can be used for conventional studies of pathogenesis and therapy as long as the modified pathogen retains the virulence of the parental strain. Moreover, this has led to an increased number of reports concerning genetically modified parasites that express bioluminescent and/or fluorescent reporters. This was principally developed for *in-vitro* infection studies and to monitor diseases in living animals [2], [3]. Bioluminescent pathogens expressing the sea pansy *Renilla reniformis* luciferase have been used in experimental murine infections of *Toxoplasma gondii* [4] as well as in the rodent malaria parasite *Plasmodium berghei* [5]. A recent study has allowed scientists to identify the liver stages of firefly luciferase-expressing parasites in living animals [6].

This approach has also been successfully implemented in trypanosomatids. Lang and co-workers showed that a luciferase expressing *L. amazonensis* strain was useful for rapid screening of drugs in infected macrophages [7]. Further studies used the same techniques with *L. major* [8], *L. infantum* [9] and in *in-vivo* murine experimental infections [7], [10]. Besides, the use of *Trypanosoma brucei* expressing *R. reniformis luc* gene has permitted scientists to find unusual colonizing niches during the progression of African trypanosomiasis [11].

Fluorescent imaging offers several benefits: i) unlike light-emitting proteins, fluorescent reporters do not require specific substrates: ii) the fluorescence emitted is very stable over time and iii) this approach is useful when studying tissue harvested from infected animals since parasites can be individually identified [12].

The first transgenic *Leishmania* species expressing the green fluorescent protein (GFP) was reported by Beverley's group [13]. Episomally transfected *Leishmania* spp. with GFP or enhanced GFP (EGFP) have enhanced High Throughput Screening (HTS) methods in free-living promastigotes [14]–[17] and amastigotes [18]–[22]. However, only recently, the stable transfection of the EGFP reporter has been found suitable for both *in vitro* and *in vivo* infection studies [21]–[24]. Although native GFP produces significant fluorescence and is extremely stable, the excitation maximum is close to the ultraviolet range, which can result in damaging living cells.

Red-fluorescence labelled parasites have been used to determine the early stages of CL pathogeny at the infection site (revised by Millington and co-workers [25]). By combining a *L. major* Red Fluorescent Protein 1 (RFP)-expressing strain and dynamic intravital microscopy, the site of sandfly bites has been identified *in vivo* in a mouse model. The study reveals an essential role for both neutrophils and dendritic cells that converge at localized sites of acute inflammation in the skin following pathogen deposition [26], [27]. Using mCherry-*L. infantum chagasi* – responsible of visceral leishmaniasis in the New World – researchers have been able to report the recruitment of neutrophils and their role in non-ulcerative forms of leishmaniasis [28]. In addition, to study the mechanism regulating dentritic cell recruitment and activation in susceptible BALB/c [29] and resistant C57BL/6 mice [30] DsRed labelled parasites were used

Fluorescent parasites have been used to explain some aspects of *Leishmania* biology. *L. donovani* lines stably expressing either EGFP or RFP have been used to identify hybrid parasites produced during the early development of the sandfly [31]. In addition, a *L. major* strain, which episomally expressed the DsRed protein, was used for quantifying the infectious dosage transmitted by a sandfly bite [32].

Based on the improved photostability as well as suitability for intravital imaging, mCherry was considered the best choice for our studies in comparison to other red fluorescent proteins [33]. mCherry is a protein derived from the coral *Discosoma striata* RFP. It has a maximum emission peak at 610 nm with a 587 nm excitation wavelength. Despite the fact that it is 50% less bright than EGFP, it is more photostable and it has higher tissue penetration [12]. Because of this, it is the best-suited choice in applications of single-molecule fluorescence or multicolour fluorescent imaging [34]. In this report we describe the use of a stably mCherry-transfected *L. major* strain as a valuable tool to both *in vitro* assays for drug screening and *in vivo* pre-clinical vaccine studies in real-time.

## Materials and Methods

### Mice and parasites

The animal research described in this manuscript complied with Spanish (Ley 32/2007) and European Union Legislation (2010/63/UE). The used protocols were approved by the Animal Care Committee of the Centro de Biología Molecular and Universidad Autónoma de Madrid (Spain).

Female BALB/c mice (6–8 week old) were purchased from Harlan Interfauna Iberica S.A. (Barcelona, Spain) and maintained in specific-pathogen-free facilities for this study.

*L. major* LV39c5 (RHO/SU/59/P) strain was used for generating mCherry transgenic promastigotes. Parasites were cultured at 26°C in M199 supplemented with 25 mM HEPES pH 7.2, 0.1 mM adenine, 0.0005% (w/v) hemin, 2 µg/ml biopterin, 0.0001% (w/v) biotin, 10% (v/v) heat-inactivated foetal calf serum (FCS) and antibiotic cocktail (50 U/ml penicillin, 50 µg/ml streptomycin). Attenuated Δhsp70-II (*Δhsp70*-II::*NEO*/*Δhsp70*-II::*HYG*), used as candidate vaccine [35], is a null mutant for the *hsp70-type-II* gene, generated by targeted deletion in *L. infantum* (MCAN/ES/96/BCN150) strain [36]. Δhsp70-II promastigotes were grown in RPMI 1640 (Sigma-Aldrich) culture medium supplemented with 10% (v/v) FCS, 50 U/ml penicillin and 50 µg/ml streptomycin.

### Generation of a mCherry+*L. major* strain

The 711-bp *mCherry* coding region was amplified by PCR from pRSETb-mCherry vector, a kindly gift from Dr Roger Y. Tsien – Departments of Pharmacology and Chemistry & Biochemistry, UCSD (USA) – [37] with the primers RBF634 and RBF600 (Table 1). PCR product was cut with appropriate restriction enzymes and ligated into the *Bgl*II and *Not*I sites of the pLEXSY-hyg2 expression vector (Jena Bioscience GmbH, Germany). Parasites expressing *mCherry* Open Reading Frame (ORF) were obtained by transfection of *L. major* with the large *Swa*I targeting fragment derived from pLEXSY-mCherry by electroporation and subsequent plating on semisolid media containing 200 µg/ml hygromycin B (Sigma-Aldrich) as previously described [38]. Correct integration of *mCherry* ORF into the 18S rRNA *locus* of the resulting transgenic clones (mCherry+*L. major*) was confirmed by Southern blot and PCR amplification analyses, using the primers of Table 1. The fluorescent of stable-transfected mCherry clones was confirmed by both flow cytometry (BD FACSCantoII) and confocal microscopy (Nikon Eclipse TE2000E).

**Table 1:** Oligonucleotides used in this work.

Oligo No.		Sequence^a^^,^ b	Purposed^c^	
RBF 634	(7)	ccgCTCGAGgaAGATCT **CCACC**ATGGTGAGCAAGGGCG	mCherry	F
RBF 600	(8)	ataagaatGCGGCCGCTTACTTGTACAGCTCGTCCATGC	mCherry	R
RBF 630	(1)	CTTGTTTCAAGGACTTAGCCATG	5′integration	F
RBF 637	(2)	TATTCGTTGTCAGATGGCGCAC	5′integration	R
RBF 644	(3)	CATGTGCAGCTCCTCCCTTTC	3′integration	F
RBF 645	(4)	CCTTGTTACGACTTTTGCTTC	3′integration	R
RBF 646	(5)	ATGAAAAAGCCTGAACTCACC	HYG	F
RBF 647	(6)	CTATTCCTTTGCCCTCGGAC	HYG	R
RBF 630	(9)	CTTGTTTCAAGGACTTAGCCATG	Southern probe	F
RBF 631	(10)	GCGGAAACCGCAAGATTTTTGC	Southern probe	R

a Underlined sequence indicates restriction site.

b Bold sequence indicates optimized translation initiation sequence.

c Orientation of primers: F, forward; R, reverse.

### *In vitro* infections and drug screening

Starch-elicited peritoneal macrophages were recovered from BALB/c mice and then 5×10^4^ cells were plated on black 96 wells plates with clear bottom. Macrophages were infected at a ratio of five metacyclic promastigotes per macrophage. Metacyclic mCherry+*L. major* promastigotes were isolated from stationary cultures (4–5 days old) by Ficoll gradient centrifugation [39]. Briefly, 2 ml of parasite suspension in M199 containing approximately 7×10^7^ stationary-phase promastigotes were layered onto a discontinuous density gradient in a 15 ml conical tube consisting of 2 ml of 20% (w/v) Ficoll stock solution made in distilled water and 2 ml of 10% (w/v) Ficoll diluted in M199 medium.

Metacyclic parasites were opsonized with 4% (v/v) C5^−^ mouse deficient serum (The Jackson Laboratory, USA) at 37°C for 30 min and resuspended in RPMI containing 10% (v/v) FCS [40]. The infection was synchronized by centrifugation (330×g, 3 min at 4°C) and infected macrophages were incubated at 37°C in a humidified 5% CO_2_ atmosphere [41]. Cells were washed extensively with phosphate buffer saline (PBS) to remove the free parasites and overlaid with fresh medium, which was replaced daily thereafter. After one day incubation, to allow differentiation into amastigotes, drugs (miltefosine, amphotericin B and paromomycin) were added to the appropriate wells in a threefold dilution series in RPMI (Sigma-Aldrich) with 10% (v/v) FCS and cells were further incubated at 37°C for a further incubation of 72 h. Plates were read in a fluorescence microplate reader (Synergy HT; BioTek) (λ_ex_ = 587 nm; λ_em_ = 610 nm).

### *In vivo* infections

Metacyclic promastigotes were isolated from stationary cultures (4–5 days old) by negative selection with peanut agglutinin for mouse infections. Briefly, promastigotes were resuspended in PBS at 10^8^ cells/ml, and peanut agglutinin (Vector laboratories) was added at 50 µg/ml; the sample was incubated for 25 min at room temperature. After centrifugation at 200×g for 10 min, the supernatant contained the non-agglutinated metacyclic promastigotes [42].

The virulence of *L. major* parasites was maintained by passage in BALB/c mice by injecting hind footpads with 10^6^ stationary-phase parasites. After 6–8 weeks, animals were euthanized and popliteal lymph nodes were dissected, mechanically dissociated, homogenized and filtered. *L. major* amastigotes were isolated from murine lymph nodes by passing the tissue through a wire mesh followed by disrupting the cells sequentially through 25G_1/2_ and 27G_1/2_ needles, and polycarbonate membrane filters with pore size of 8, 5 and 3 µm (Isopore, Millipore) [43]. Isolated amastigotes were transformed to promastigote forms by culturing at 26°C in Schneider's medium (Gibco, BRL, Grand Island, NY, USA) supplemented with 20% (v/v) FCS, 100 U/ml penicillin and 100 µg/ml streptomycin. For infections, amastigote-derived promastigotes with less than five passages *in vitro* were used.

BALB/c mice were injected with several inocula (10^4^, 10^5^ and 10^6^ promastigotes/mouse) during the setting up of the model. For protection studies mice were vaccinated intravenously (tail-vein injection) with the Δhsp70-II mutant strain (2×10^7^ promastigotes/mouse) [35], or injection of PBS (control group), and four weeks post-vaccination, were infected with 2×10^5^ mCherry+*L. major* metacyclic promastigotes. The infections were performed by injection of parasites in 50 µl PBS in the right hind footpads. The growth of the lesion was monitored by fluorescence emission detection (see below). The contralateral footpad of each animal represented the negative control value. Footpad swelling was measured using a Vernier calliper and data were represented as the increment of the lesion size respect to the not infected footpad.

### *In vivo* fluorescence imaging and calibration

Fluorescence emission was measured using an intensified charged coupled device camera of the In Vivo Imaging System (IVIS 100, Xenogen). Wild type- and mCherry+*L. major*-infected animals were lightly anesthetized with 2.5–3.5% isoflurane and then reduced to 1.5–2.0%. Anesthetized animals were placed in the camera chamber, and the fluorescence signal was acquired for 3 s. Fluorescence determinations, recorded by the IVIS 100 system, were expressed as a pseudocolour on a gray background, with red colour denoting the highest intensity and blue the lowest. To quantify fluorescence, a region of interest was outlined and analyzed by using the Living Image Software Package (version 2.11, Xenogen).

### Quantification of parasite load

The total number of living parasites invading the target organs (popliteal lymph node draining the injected site) was calculated from single-cell suspensions that were obtained by homogenization of the tissue through a wire mesh. The cells were washed and cultured in Schneider's medium containing 20% (v/v) heat-inactivated FCS, 100 U/ml penicillin and 100 µg/ml streptomycin. The cell suspensions were serially diluted and dispensed into 96-well plates. The plates were incubated for 10 days and then each well was examined and classified as positive or negative according to whether or not viable promastigotes were present. The number of parasites was calculated as follows: Limit Dilution Assay Units (LDAU) = (geometric mean of titer from quadruplicate cultures)×(reciprocal fraction of the homogenized organ added to the first well). The titer was the reciprocal of the last dilution in which parasites were observed [44].

## Results

### Generation of a functionally fluorescent *L. major* strain

**Figure 1:**
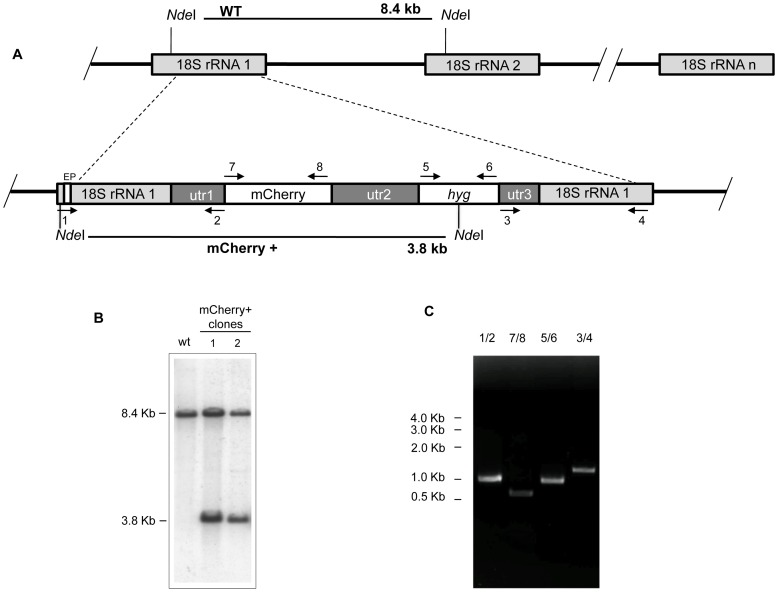
Strategy of integration of *mCherry* encoding gene into genomic rRNA *locus* of *L. major*. A) Scheme of the structure of the 18S rRNA locus on wild type and planned integration of *mCherry* gene. Key: utr1: 5′non-translated region of *aprt* gene; utr2: 1.4 kb intergenic region from *cam* operon; and utr3: 5′UTR of *dhfr*-*ts* gene; *hyg*; hygromycin B resistance cassette. The narrow white box on the left side corresponds to the external probe (EP) used for southern blot analyses. B) Southern blot analysis of two *L. major* clones after selection with hygromycin B. DNA was digested with *Nde*I and hybridized with the labelled EP. The similar intensities of 8.4 and 3.8 kbp bands could be due to inefficiency transfer of the large band to the membrane. C) PCR confirmation of successful integration of the reporter cassette. Primers 1/2 and 3/4 together confirm the correct integration of the reporter cassette from the 5′ and 3′ sides, respectively. Primers 5/6 confirm the presence of the HYG marker, and primers 7/8, 9/10, confirm the presence of *mCherry* ORF, respectively. Primers: 1(RBF630); 2(RBF637); 3(RBF644); 4(RBF645); 5(RBF646); 6(RBF647); 7(RBF634) and 8(RBF600) (see Table 1 for sequences).

Aimed to create a *L. major* fluorescent strain we electroporated wild-type promastigotes with the lineal 5874 bp *Swa*I-*Swa*I fragment containing the ORF encoding *mCherry* as well as the *hyg* selection marker of the pLEXSY-mCherry plasmid. After selection on semisolid plates containing 100 µg/ml hygromycin B, individual colonies were seeded in M199 liquid medium supplemented with 10% FCS and hygromycin B. Genomic DNA isolated from these cultures was used to confirm the correct integration of the target sequence into the 18S rRNA locus of *L. major* genome. Figure 1A shows a schematic representation of the planned integration. Genomic DNA from wild-type strain and two hygromycin B resistant clones were digested with *Nde*I and hybridized with a labelled external probe (EP). As shown in the Southern analysis of Fig. 1B, wild-type DNA digested with *Nde*I yielded an 8.4-kb hybridization band, whereas in the two-hygromycin B resistant clones an additional 3.8-kb hybridization band was observed; this band is generated by the integration event (Fig. 1A) corresponding to the expected size. Further confirmation of the correct planned replacements was confirmed by PCR (Fig. 1C) using the set of primers depicted in Fig. 1A.

**Figure 2:**
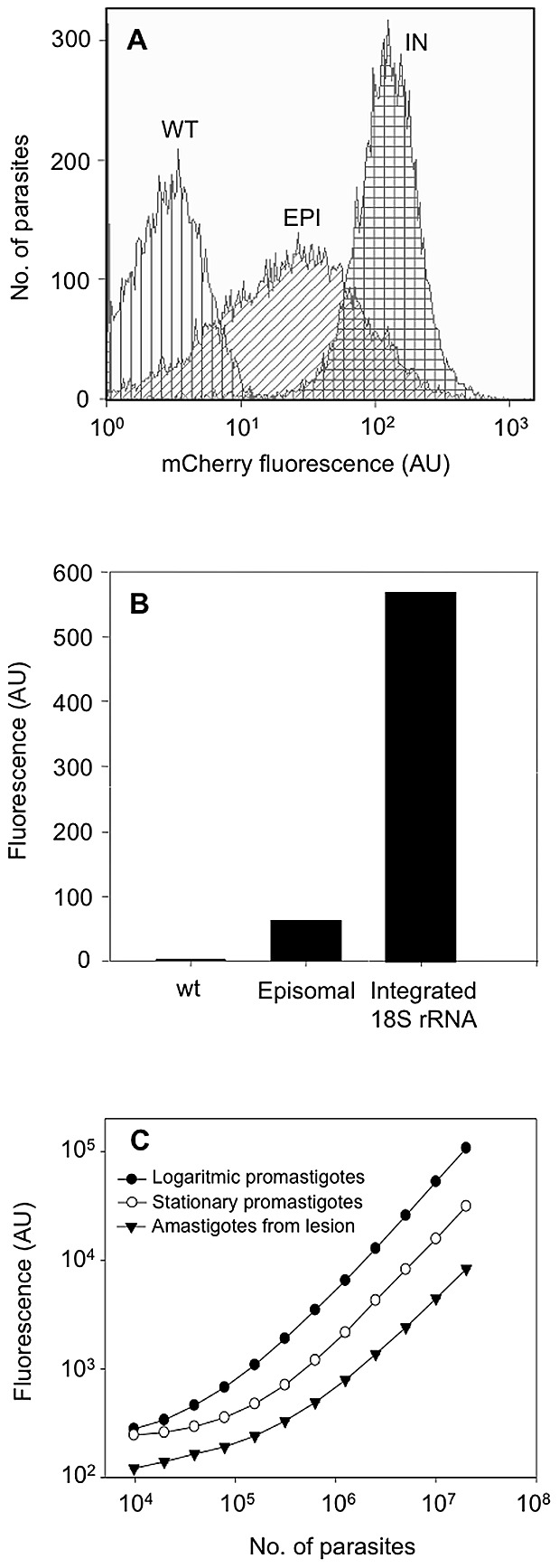
*mCherry* gene is functionally expressed in *L. major* parasites. Wild type (WT), pLEXSY episomal- (EPI) and integrated- (IN) *mCherry* expressing promastigotes were grown in the presence of hygromycin B and fluorescence levels were measured by flow cytometry. A) Histogram plot representative of the distribution of mCherry fluorescence levels in different populations of cells. B) Mean fluorescence intensity emitted by WT and the engineered parasite strains. The fluorescence is expressed as arbitrary units (AU). C) Correlation between fluorescence signal and the number of logarithmic promastigotes (•), metacyclic promastigotes (○) and freshly isolated amastigotes (▴) of mCherry+*L. major*. Two-fold serial dilutions were applied and parasites were counted using a Coulter counter.

The mCherry expression in stable-transfected *L. major* promastigotes (mCherry+*L. major*) was monitored by flow cytometry. Cell populations of mCherry+*L. major* strain and a parasite line containing the pLEXSY-mCherry episome emitted strong red fluorescence when they were excited at wavelength of 587 nm (Fig. 2A). Clones with integrated *mCherry* gene had an average 10-fold higher fluorescence than the ones expressing the gene episomally. This is an expected result given that the *mCherry* gene was integrated under the control of rRNA promoter, which is known to present high-level transcription rates. As shown in Fig. 2B, both strains (episomal and integrative) were strongly more fluorescent than untransfected parasites.

In order to establish the correlation between parasite number and fluorescence intensity, different number of procyclic and metacyclic promastigotes as well as freshly isolated amastigotes from infected animals were placed in 96-well plates and their fluorescence intensity was measured spectrofluorometrically. A clear correlation between fluorescence intensity and the number of the three parasitic forms was observed (Fig. 2C).

The stability of mCherry expression was monitored over a period of 6 months after transfection and no change was observed in fluorescence intensity during this period, even in the absence of hygromycin B.

### *In vitro* infections with mCherry+*L. major* parasites

**Figure 3:**
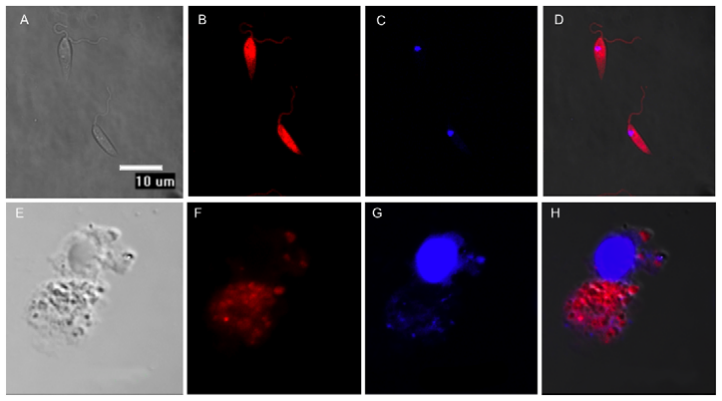
Fluorescent detection of mCherry+*L. major* transfected parasites by confocal microscopy. Top panel (A, B, C, D) *L. major* promastigotes. Bottom panel (E, F, G, H) BALB/c mouse peritoneal macrophages experimentally infected with mCherry+*L. major* metacyclic promastigotes. (A, E) Differential Interference Contrast (DIC); (B, F) mCherry+*L. major* emitting red fluorescence; (C, G) DAPI staining of nucleic acids; (D, H) merged images. The microscopy images were acquired with a Nikon Eclipse TE2000E confocal microscope.

**Figure 4:**
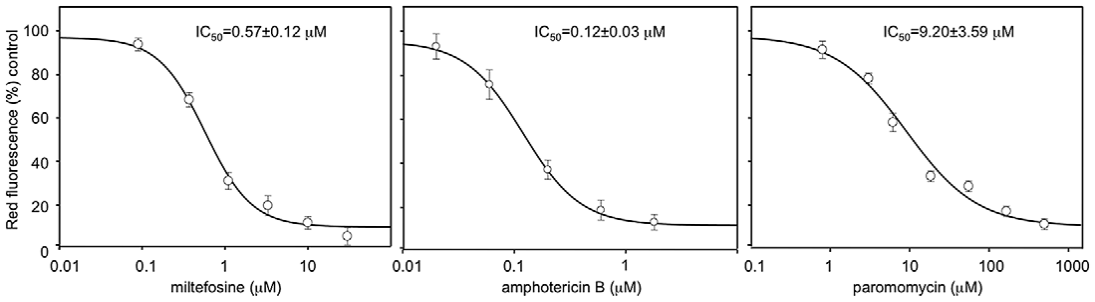
IC_50_ calculation after a 72-h period of exposure to currently used leishmanicidal drugs in infected peritoneal mouse macrophages with mCherry+*L. major*. Drugs were added in a threefold dilution series, being the highest concentration 100, 2 and 500 µM for miltefosine, amphotericine B and paromomycin, respectively. IC_50_ values were calculated from dose-response curves performed in triplicate and repeated twice after nonlinear fitting with the SigmaPlot program.

Once the infectivity of the mCherry+*L. major* parasites was recovered through mouse infections, the amastigotes obtained from cutaneous lesions were differentiated back into promastigotes. These cells were grown up to stationary phase and used to infect freshly isolated BALB/c peritoneal macrophages at a 5∶1 multiplicity in 24-well plates. Figure 3 shows fluorescence images of either promastigotes or amastigotes internalized in macrophages. A strong red fluorescence emission from free-living mCherry+*L. major* promastigotes was observed by confocal microscopy (Fig. 3B). Similarly, round-shaped red fluorescent emitting amastigotes were observed inside parasitophorous vacuoles in the cytoplasm of the infected macrophages (Fig. 3F). The course of the *in vitro* infection was followed over a period of 48 h by measuring the absolute fluorescence of the infection and the percentage of infected macrophages. These experiments were carried out in parallel with others using the classical Giemsa staining to determine parasite load *in vitro* (data not shown). No differences between both methods were accounted thus pointing to the suitability of fluorescence analyses to assess the infectivity of mCherry+*L. major* strain on mouse macrophages. A major application of a fluorescent *Leishmania* model would be its usefulness to perform HTS of potential leishmanicidal compounds *in vitro*. To assess the suitability of our mCherry+*L. major* for this goal, current drugs used in the treatment of human leishmaniasis (miltefosine, paromomycin and amphotericin B) were assayed in *Leishmania-*macrophage infections at different concentrations over a 72 h-span. Absolute fluorescence emitted by mCherry+*L. major* infected macrophages was plotted *vs*. drug concentrations, obtaining the dose-response curves of Fig. 4. Nonlinear regression analysis of the curves, fitted by the SigmaPlot statistic package, reached IC_50_ values of 0.12±0.03 µM for amphotericin B, 0.57±0.12 µM for miltefosine and 9.20±3.59 µM for paromomycin. In all the cases the difference in fluorescence emission corresponded to a difference in the percentage of infected cells, also observed microscopically. These findings clearly showed that the mCherry+*L. major* strain is a useful tool for *in vitro* drug screening.

### Validation of a CL murine model with mCherry+*L. major*

In order to determine whether mCherry+*L. major* parasites could be detected *in vivo* using whole-body imaging, 10^4^, 10^5^ and 10^6^ metacyclic forms of the fluorescent-transgenic strain were injected subcutaneously into the hind footpads of six BALB/c mice per group. Lesion progression monitored both by direct measuring of fluorescence emission by mCherry+*L. major* amastigotes (recorded in an IVIS 100) and by the development of hind-limb lesions assessed by measuring the thickness of the footpads with a Vernier calliper. Animals were examined every seven days for a total of eight weeks (except the group infected with 10^6^ parasites that were sacrificed at 6^th^ week post-infection because the appearance of ulcerations in the footpads).

**Figure 5:**
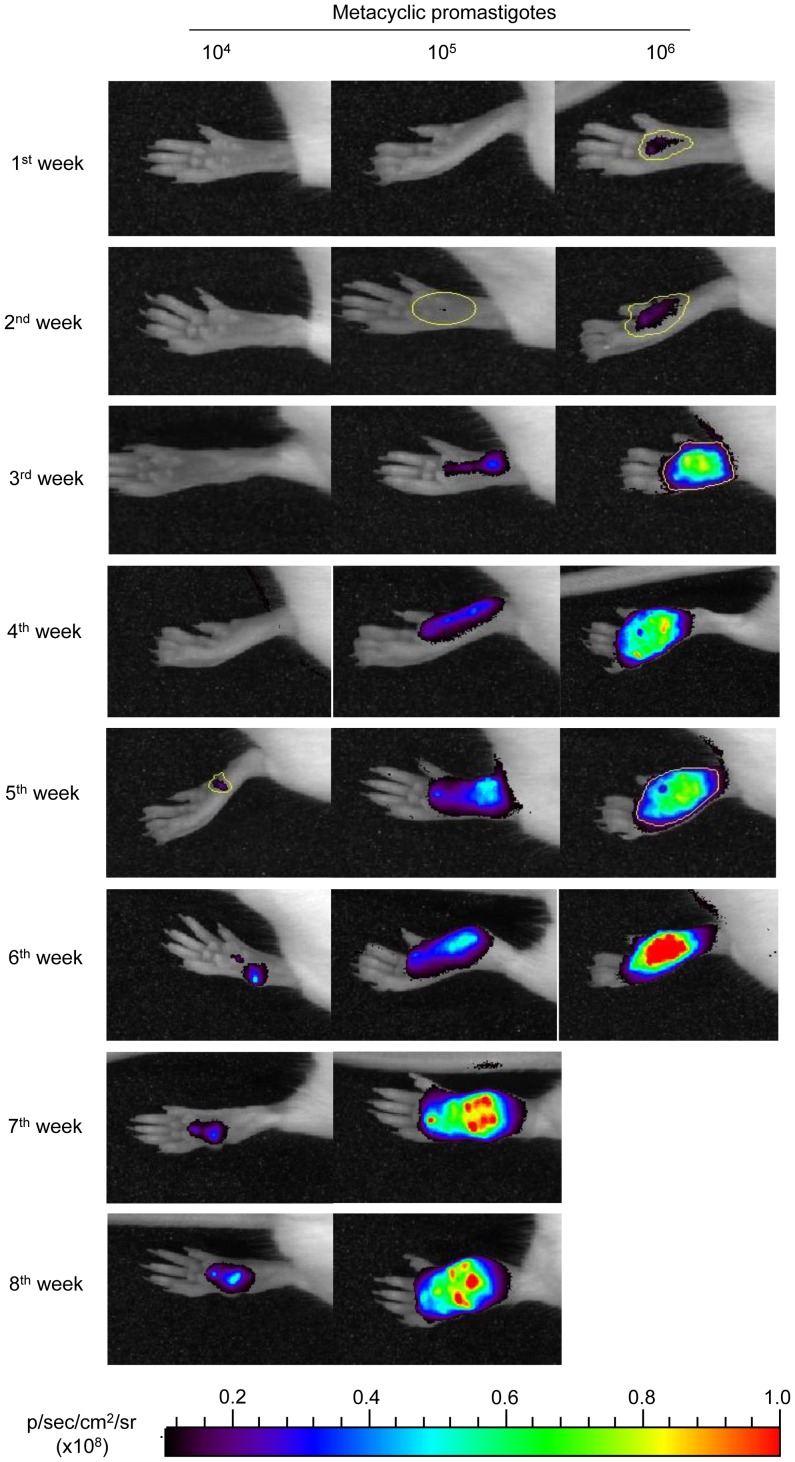
Progression of an experimental infection with mCherry+*L. major* in BALB/c mice. Photographs of mouse footpads over the time after inoculation with 10^4^; 10^5^ and 10^6^ mCherry+*L. major* metacyclic promastigotes. The images were taken weekly using an In Vivo Imaging System (IVIS 100; Xenogen) device. Six mice per dose were used in this experiment, and one representative mouse was chosen for all of the photographs. Examples of Regions of Interests (ROIs) used for quantification are marked in yellow.

**Figure 6:**
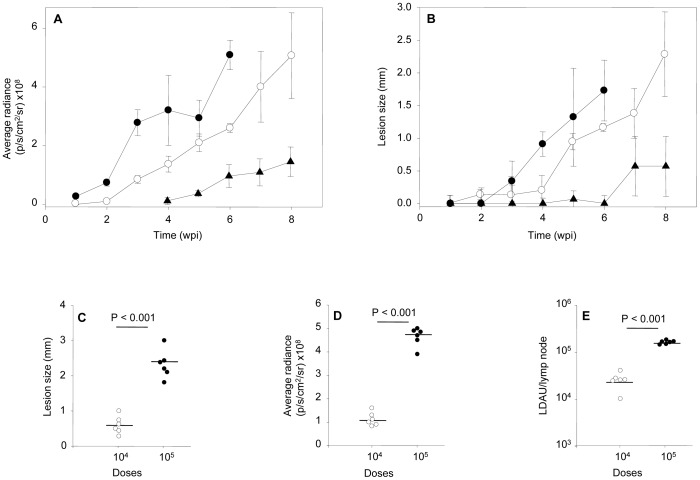
Follow up of the *in vivo Leishmania major* infection. A) Plot comparing the progression of fluorescence signal (pixel/second/cm^2^/sr) mean ± SEM over the time. (B) Plot comparing the progression of lesion size (mm) mean ± SEM over the time. Key: infective inocula: 10^6^ (•), 10^5^ (○), 10^4^ (▴) metacyclic promastigotes per mouse. Effect of infective inocula on: C) lesion size (mm), D) fluorescence signal (pixel/second/cm^2^/sr), E) parasitic load (LDAU) in the poplyteal lymph node draining the lesion of infected animals. Key: infective inocula: 10^4^ (○), 10^5^ (•). Data were individually collected at the end of the experiment.

Figure 5 shows the fluorescence intensity recorded weekly from the footpads of representative mice of each inoculum group (10^4^, 10^5^ and 10^6^ metacyclic promastigotes per mouse). The fluorescent signal (estimated as average radiance: p/s/cm^2^/sr) was plotted against the infection time of each inoculum (Fig. 6A). Fluorescent signal was detected after the first week post-infection in mice infected with 10^6^ metacyclic parasites (radiance = 0.26×10^8^ p/s/cm^2^/sr), reaching a radiance of 5.0×10^8^ p/s/cm^2^/sr five weeks later. In the group of mice injected with 10^5^ metacyclic parasites, the fluorescence was detected the third week after inoculation (radiance = 0.8×10^8^ p/s/cm^2^/sr), reaching similar intensity than the mice group injected with 10^6^ parasites at the 8^th^ week of inoculation. Finally, the fluorescence signal of mice injected with 10^4^ metacyclic mCherry+*L. major* parasites was not detectable until the 5^th^ week (radiance = 0.35×10^8^ p/s/cm^2^/sr), reaching the maximum intensity (radiance = 1.14×10^8^ p/s/cm^2^/sr) at the end of the experiment.

The success of infection defined as the lesion onset and its development in the inoculated footpads over the time, was observed in every mice. Although there was a good correlation to lesion size, fluorescence was more sensitive to evaluate the progression of infection. Figure 6B shows that lesion emergence was dependent on the size of pathogen inoculum and it was hardly measurable during the first weeks after infection. Lesion size in mm was 0.34, 0.94 and 0.57 measured at the third, fifth and seventh week, respectively corresponding to 10^6^, 10^5^ and 10^4^ metacyclic promastigotes per inoculum. It is remarkable that a weak but measurable fluorescence signal from infected hind limbs was detectable two weeks prior to visible and measurable injury took place in all dose groups.

As expected and since BALB/c mice have a predisposition to develop an anti-inflammatory Th2 response, the lesions appearing after infection setup were non-healing without treatment. A correlation analysis of the fluorescence emitted by the lesions toward the end of the 8-week period from mice infected with 10^4^ and 10^5^ metacyclic mCherry+*L. major* parasites (Fig. 6C) shows significant differences between both groups (P<0.001) using unpaired t-Student test. These differences were also found when the lesion thickness of the footpads was compared using the traditional calliper-based method (Fig. 6D). There was a clear correlation between both parameters in both dosing groups with an estimated Pearson coefficient of 0.94. At the end of the experiments, animals were sacrificed and the popliteal lymph nodes draining the lesions were dissected under sterile conditions. The parasite load of these organs was determined by the limit dilution method. Figure 6E shows the number of promastigote-transforming amastigotes estimated in the animals of both dosing groups, showing significant differences (P<0.001) and correlating highly with both size lesion and fluorescence (Pearson coefficient = 0.79).

### Application of mCherry+*L. major* to a model of murine vaccination

The suitability of this *in vivo* approach was assessed for the evaluation of an experimental vaccination protocol against CL that had been previously shown to be effective on a *L. major*–BALB/c infection model [35]. In previous studies, it was established that intravenous inoculation with *Leishmania* promastigotes, lacking both alleles of the *hsp70-*II gene (Δhsp70-II line), confers a partial protection against *L. major* infection in mice. For this study, we inoculated a group of six mice with 2×10^7^ promastigotes of Δhsp70-II mutant and four weeks later, mice were challenged with 2×10^5^ metacyclic forms of the mCherry+*L. major* strain into mouse footpads. In parallel to the vaccinated group, a control group was injected with the same inoculum of mCherry-expressing transgenic parasites.

**Figure 7:**
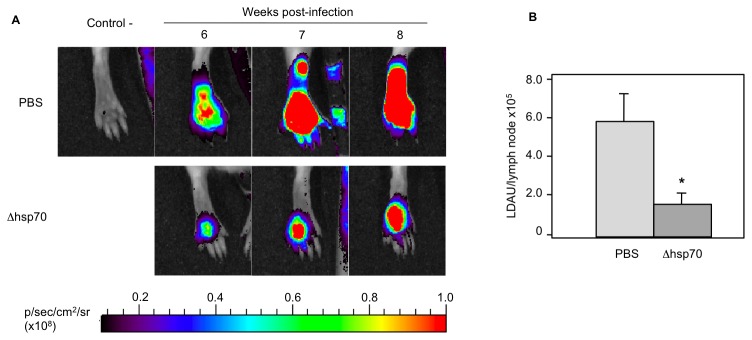
Effect of vaccination with Δhsp70-II Leishmania mutant on development of a BALB/c CL model produced by mCherry+*L. major* strain. A) Top panel shows a non-infected animal (negative control) and the fluorescent signal emitted by hind footpads of animals infected with 2×10^5^ metacyclic mCherry+*L. major* promastigotes at different time points. Bottom panel shows the footpads of animals immunized by intravenous administration of 10^7^ Δhsp70-II metacyclic promastigotes per mouse four weeks before the challenge. Six mice in each group were imaged weekly. B) Parasite burdens in popliteal lymph nodes mean (LDAU/lymph node×10^5^) ± SEM of six animals. Statistical differences were observed between groups; *, *P*<0.01 using de paired Student *t* test.

Red fluorescence emission in the footpad of mice infected with mCherry+*L. major* parasites was followed over the time in both groups (Fig. 7A). Fluorescence signal was detected in both groups four weeks after challenge; however, fluorescence signal was higher in control group mice than in vaccinated animals. By the end of the 8^th^ week animals were euthanized, the poplyteal lymph nodes dissected, homogenized and the parasite load determined as above. Figure 7B shows an 80% reduction (P<0.001) in the parasite load of popliteal lymph nodes of vaccinated group related to the control group. Therefore, since reproducible results were obtained with both parasite quantification and fluorescence emission methods, we conclude that the murine model of CL established with the mCherry+*L. major* fluorescent strain might be a suitable system for testing antileishmanial therapies both *in vitro* and *in vivo*.

## Discussion

Transgenic parasites expressing reporter proteins are valuable tools to perform robust HTS platforms [45] and to understand the underlying mechanisms of pathogenesis [3]. GFP is one of the most commonly used reporters among fluorescent proteins. Several mutants derived from native GFP have been developed to cover longer wavelengths of the spectrum. Reporter molecules, whose emission peak is in the red spectral range, the same as mCherry, are excellent candidates for these kinds of studies. Furthermore, light absorption by tissues in the red and far-red spectra is reduced and consequently, the penetration is higher [46]. Moreover, mCherry is the best general-purpose red monomer due to its superior photostability compared to mStrawberry and DsRed, which is inadequately folded at 37°C [13].

The integration of the reporter gene into the 18S rRNA locus of *L. major* represents an efficient and effective strategy to guarantee a stable expression when the parasites need to be grown in the absence of selection drugs for both *in vitro* screenings and in mice infections [7], [22]–[25], [47], [48].

mCherry fluorescence was detected in the different stages of the *L. major* life cycle. Lesion-derived amastigotes showed two times less activity than metacyclic promastigotes. In turn, these were three times less fluorescent than logarithmic promastigotes. Similar results were reported in promastigotes of different *Leishmania* species, in which luciferase expression was much higher than that of amastigotes from animal lesions and experimentally infected macrophages, respectively [7], [47]. However, Mißlitz and co-workers [23] showed that EGFP expression levels were 2–10 times higher in amastigotes than in promastigotes of both *L. mexicana* and *L. major*. Although these species were stably transfected by the integration into the 18S rRNA locus; they differed in the downstream region of the reporter gene. Whilst no specific 3′ untranslated region implicated in the stage-specific regulation was included downstream on the *luc* gene [47]; the intergenic calmodulin A region was configured into the pLEXSY plasmid ([7] and the present work). In a similar way, the cysteine proteinase intergenic region (cpb2.8) was included in the studies conducted by Mißlitz [23]. Intergenic sequences responsible for a high transcription rate in amastigotes should be included in future vectors for regulating the reporter's expression. In this sense, technologies such as RNA sequencing can provide a complete transcriptome that could be used to improve the expression technology in both promastigote and amastigote forms [49], [50].

Assays designed to simplify rapid and large-scale drug screenings are not performed on the clinically relevant parasite stage, but on promastigotes instead. Axenic amastigotes have also been screened by means of HTS platforms [9], [51], [52]. However, expression arrays comparing both axenic amastigotes and those isolated from infected macrophages have shown metabolic differences, impaired intracellular transport and altered response to oxidative stress [53].

The suitability of mCherry+*L. major* transgenic strain is an important tool for bulk testing of drugs in the intracellular amastigote stage. This was demonstrated further by using three drugs in clinical use against leishmaniasis: amphotericin B, paromomycin and miltefosine.

Most of the drug screening assays attempted to analyze intracellular parasites using GFP-tranfected *Leishmania* spp. Theses methodologies clearly showed that there was not enough sensitivity to enable a precise and reliable microplate screening. Consequently an in-depth flow cytometric analysis is required [54]. Recently, a novel method for assessing the activity of potential leishmanicidal compounds on intracellular amastigotes through the use of resazurin (a fluorescent dye with emission wavelength in the red spectrum) has advanced to microplate analysis [55]. Unlike the GFP-expressing parasites, mCherry emission is also found in the same spectral range as resazurin. This level of sensitivity was sufficient to detect 10^4^ amastigotes isolated from lesions. This means that mCherry reporter provides several benefits over fluorescent proteins for performing HTS into microplate format.

Other advantages of fluorescent proteins are that they allow a dynamic follow up (kinetic monitoring) of the drug efficiency using a single plate. Drugs must be maintained in the culture medium for a time long enough for them to take effect. On the contrary, multiple plates are required if a specific substrate is added, requiring one for each recorded time interval.

Through our research we want to raise the importance of the source of host cells used for experimental infections when drug-screening assays are carried out. Several differences in the host-parasite interactions have been pinpointed when comparing primary macrophages with immortalized human macrophage-like cell lines [56]. Most of the current multiwell-screening methods involve established-macrophage cell lines since it is quite difficult to scale-up a procedure based on primary macrophages [57], [18], [58], [20], [22], [59]. Accordingly, a well-planned combination of different approaches (promastigote/intracellular; cell line/primary cultures) would help us to identify lead compounds through large-scale drug screening [60], [61].

The manipulation of large numbers of potential drugs not only requires easy-to-use, repeatable and readily quantifiable tests, but also it needs to mimic natural conditions within the host cell. Because of the profound influence of the host's immune response on the treatment of leishmaniasis, new approaches should include the whole immunopathological environment found at the host-parasite interaction site. However, only one alternative approach has been used in order to transfer this immunological concept to HTS systems [43], [61].

The main advantages of mCherry-transfected parasites are automation and miniaturization. As experiments are performed in 96-well plates, reducing costs of reagents, and time of analysis is of great importance. Besides, we can also eliminate tedious steps such as staining or cell lysis. In addition this allows a dynamic follow up as cells remain viable after each reading time interval.

As the stable integration of the gene encoding reporter proteins represents a valuable tool for assessing whole-body imaging in laboratory mice [47], [7], [10], [62]–[64], we decided to use the same mCherry-transfected strain for *in vivo* applications. Experimental infections with *L. major* in BALB/c mice footpads resulted in a non-healing and destructive chronic lesion at the site of injection. The mCherry *in vivo* model developed in this study clearly allowed the fluorescence signal in the first week post-inoculation with 1×10^6^ stationary parasites, a dose used in leishmanial research to induce the rapid development of CL. Similar models in BALB/c mice with EGFP, used 10- and 200-fold parasite doses and the fluorescence signal was visualized afterwards [21], [24]. The lymph node draining the lesion was not detected in this study, probably because the lower inoculum used or because of the shorter time of testing when the animals were killed. Previously, reports detected the fluorescence or luminescence signal emitted by the lymph node a long time after post-infection (2.5–10 months) [7], [21], [23].

In order to evaluate the eligibility of our fluorescent tool for the monitoring of *in vivo* treatments, we applied this approach by evaluating an experimental vaccine against leishmaniasis that had been previously shown to be effective. Our previous studies showed that a *L. infantum* strain lacking the *hsp70-II* gene (Δhsp70-II line) conferred resistance to a subsequent infection with *L. major* [35], [36]. We found that the progression of the infection was efficiently and effectively observed by recording the mCherry signal through real-time imaging. Vaccination of infected mice for a period of 8 weeks with the vaccine reduced the infection when compared with the control group. Further to this, Mehta and co-workers successfully used a similar vaccination approach to assess the efficiency of a real-time imaging platform using an engineered strain of *L. amazonensis* expressing the *egfp* gene [24].

In conclusion, we have developed a valuable fluorescence-emitting *L. major* transfected strain. This strain allows us: i) actual imaging, which is important when studying tissue harvested from an infected animal because parasites can be individually identified; ii) to easily develop new, fast and efficient platforms for the screening of potential leishmanicide drugs testing thousands of compounds in *Leishmania* amastigote-infected macrophages; iii) to reproduce the infection in real-time due to the virulence of *L. major*-transfected strain, which in turn increases the sensitivity of detection especially at the earlier phases of the process. Furthermore, this avoids the unnecessary slaughter of large amounts of animals at different time-points owing to direct imaging and fluorescence testing, which can be performed without traumatic handling to the animals.
